# Community-based intervention for managing hypertension and diabetes in rural Bangladesh

**DOI:** 10.1186/s41182-023-00574-0

**Published:** 2024-01-24

**Authors:** Yurie Kobashi, Syed Emdadul Haque, Kayako Sakisaka, Isamu Amir, Megumi Kaneko, Mahmuda Mutahara, Sanzida Mubassara, Abul Kashem, Masaharu Tsubokura

**Affiliations:** 1 Department of Radiation Health Management, Fukushima Medical University School of Medicine, Fukushima City, Fukushima, 960–1295 Japan; 2 Global Exchange Center, Fukushima Medical University School of Medicine, Fukushima City, Fukushima, Japan; 3 Health Equity Research Institute, Abiko City, Chiba, Japan; 4 UChicago Research Bangladesh, Dhaka, 1230 Bangladesh; 5 Faculty of International Liberal Arts, Kaichi International University, Kashiwa-Shi, Chiba, Japan; 6 Bridge of Community Development Foundation, Narail, Bangladesh; 7 Department of Botany, Jahangirnagar University, Savar Union, 1342 Bangladesh

**Keywords:** Non-communicable diseases, Community survey, Community-based participatory research, Global health, Bangladesh

## Abstract

**Background:**

Approximately 80% of non-communicable diseases (NCDs) have been reported in low- and middle-income countries (LMICs). However, studies on the usefulness of educational interventions run by non-healthcare workers in combating NCDs in resource-limited areas in rural parts of LMICs are limited. This study aimed to identify the effectiveness of a community-based simple educational program run by non-healthcare trained staff for several outcomes associated with NCDs in a resource-limited area.

**Methods:**

Six villages in the Narail district in Bangladesh were selected, two each in the first and second intervention and the control groups, in the Narail district in Bangladesh were selected. Pre- and post-intervention survey data were collected. The first intervention group received the “strong” educational intervention that included a checklist poster on the wall, phone call messages, personalized advice papers, seminar videos, and face-to-face seminars. The second intervention group received a “weak” intervention that included only a checklist poster on the wall in their house. The outcome was the proportion of NCDs and changes in systolic blood pressure and blood sugar level. Confidential fixed-effects logistic regression and multiple linear regression were performed to identify the effectiveness of the intervention.

**Results:**

Overall, 600 participants completed the baseline survey and the follow-up survey. The mean systolic blood pressure reduced by 7.3 mm Hg (95% confidence interval [CI] 4.6–9.9) in the first intervention group, 1.9 mm Hg (95% CI − 0.5–4.2) in the second intervention group, and 4.7 mm Hg (95% CI 2.4–7.0) in the control group. Multiple linear regression analysis showed that the between-group differences in the decline in systolic blood pressure were significant for the first intervention versus control (*p* = 0.001), but not for the second intervention versus control (*p* = 0.21). The between-group differences in the reduction in blood glucose after the intervention, were not significant on multiple linear regression analysis.

**Conclusions:**

Community-based educational interventions for NCDs provided by non-healthcare staff improved the outcomes of hypertension and risk behaviors. Well-designed community-based educational interventions should be frequently implemented to reduce NCDs in rural areas of low- and middle-income countries.

*Trial registration* UMIN Clinical Trials Registry (UMIN-CTR; UMIN000050171) retrospectively registered on January 29, 2023.

**Supplementary Information:**

The online version contains supplementary material available at 10.1186/s41182-023-00574-0.

## Introduction

Non-communicable diseases (NCDs) are the leading causes of early mortality and disease burden worldwide; approximately 80% of NCDs have been reported in low- and middle-income countries (LMICs) [1, 2]. Community-based integrated services with a public health approach and collaboration of various sectors are needed to confront the risk behaviors and risk factors for NCDs in settings with limited resources [3]. Previous studies reported that community-based programs against NCDs are essential for the participation of community residents and the integrated implementation of strategies [4–6]. Community-based approaches are essential for NCDs in resource-limited settings; interventional studies to identify the effective strategies toward NCDs are urgent public health issues.

Several countermeasures have been adopted to confront the issues of NCDs and risk behaviors in various communities, such as community-based education by community health workers or health facilities [7–10], mobile-health or telehealth intervention [11–17], EMPOWER-participatory action research [18, 19], text messages [20], use of stickers/labels on salt containers [21], community health assessment programs [22], and others. Especially, educational interventions for residents were found as promising countermeasures against NCDs and risk behaviors at the community level. Moreover, professional health resources are limited in LMICs, where NCDs are becoming leading health issues; hence, the participation of non-healthcare workers is crucial to the success of these countermeasures. Nevertheless, very few studies have investigated the usefulness of educational interventions run by non-healthcare workers in combating NCDs in resource-limited areas in rural parts of LMICs.

Bangladesh, located in the Southeast Asia, is classified as an LMIC [23]. In rural areas of Bangladesh, hypertension and NCDs have been a leading cause of mortality [24]. In addition, there were many patients who were not diagnosed NCDs in rural area; they were not interested in visiting hospital with the issues of social economics, lifestyle, and luck of awareness [24]. As public hospitals and health facilities have to handle communicable diseases, maternal and child health, and severe diseases, health resources for NCD prevention are limited. Furthermore, only 23% of qualified health workers work in rural area [25]. The number of qualitied health care workers per 10,000 population was 98 in urban area, and 22 in rural area in report of 2021 [25]. To confront these issues, residents of rural communities and non-healthcare workers need to be prepared to confront the threat of NCDs; the educational interventions were required among these people. Thus, rural Bangladesh was a suitable area for investigating the effectiveness of community-based educational interventions run by non-healthcare workers to combat NCDs.

This study aimed to identify the effectiveness of a community-based, simple educational program run by non-healthcare workers in reducing the outcomes associated with NCDs.

## Methods

### Study design

**Fig. 1:**
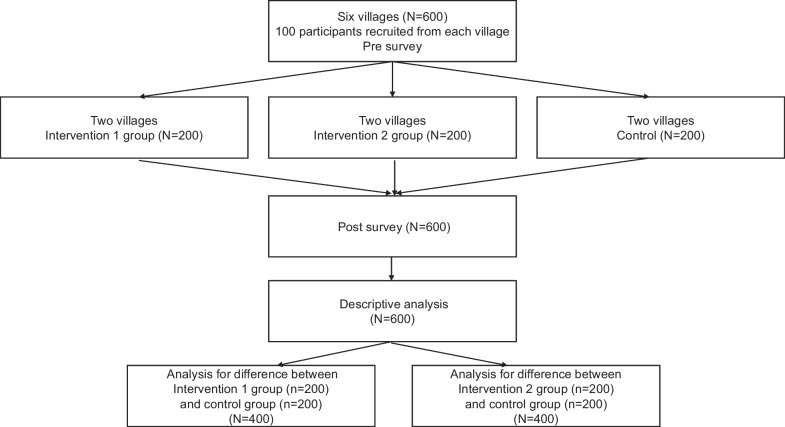
Flow diagram of the community-based intervention by non-healthcare workers for managing non-communicable diseases

This parallel interventional study included six villages (each village randomly assigned to the first and second intervention and the control condition). The villages in Lohagora Upazila of Narail district, a rural area of Bangladesh were randomly selected. The baseline survey was conducted between March 15 and June 11, 2022, and the follow-up survey was conducted between August 21 and October 16, 2022. The average duration between the pre- and post-survey was 143 days (Fig. 1). This study was conducted as part of Narail NCDs community survey.

### Participants

Residents aged between 20 and 80 years and those who agreed to participant to the present study were selected; 100 residents from each of the 6 villages were included, resulting in a sample size of 600.

### Intervention

Two non-healthcare staffs were trained to use the digital portable blood pressure monitor (Omron, Japan) and portable blood sugar measuring instrument (VivaChek Ino glucose test strip, US). They measured the blood pressure and blood glucose at the pre- and-post surveys and conveyed the result and its meaning to the participants. The staff were also trained to provide educational information using checklist posters (Additional file 1: Figure S1), phone calls, advice papers (Additional file 1: Figure S2), video seminars, and face-to-face seminars. All interventions were conducted in the local language. We selected the interventions from previous interventional research on NCDs, namely checklist poster [26], seminar and video interventions [7–10], and texts messages [20]. We experienced the project that easy paper checklist about pesticide protective behavior improved outcome in this area [26]. Nevertheless, previous literatures showed that strong interventions were required to improve the outcomes associated with NCDs. This is because we conducted two types of interventions in the present study.

The contents of the checklist poster were explained by the non-healthcare staff for participants; the participants were required to stick the poster on their house wall (Additional file 1: Figure S1). The local staff made phone calls every two weeks and explained the risk factors of NCDs and prevention methods to the participants individually. Advice papers were distributed once for personalized advice (Additional file 1: Figure S2). Seminar videos in local language, each approximately 20 min long, were provided by the local staff thrice during the study period: The staff visited each participant’s house and approximately 60% of the participants agreed to see the educational videos. The contents included information on hypertension, diabetes, and prevention method and life style toward NCDs. A total of three face-to-face seminars in local language, each approximately 30 min long, were provided by the researchers. The participants were informed about the lecture seminar in advance and they were gathered in a suitable place in each village. The contents included the information on hypertension, diabetes, and prevention method and life style toward NCDs in detail. Approximately 40% of the participants joined this seminar each time. The participants could ask questions about NCDs directly to the researchers in these seminars.

The first (strong) intervention included measurement of blood pressure and blood sugar along with feedback of the results, educational checklist wall poster, phone call in every two weeks, one personalized advice paper, several video seminars, and several face-to-face seminars. The second (weak) intervention included a checklist wall poster and measurement of blood pressure and blood sugar along with feedback of the results. The control group only underwent blood pressure and blood sugar measurements and received the feedback at the pre- and post-survey. The mean intervention duration was 158 days for the first intervention group, 143 days for the second intervention group, and 129 days for the control group, respectively. Among the six villages, two villages each were assigned randomly to the first intervention group, the second intervention group, and the control group by the researchers.

### Assessment of interventions

Trained non-healthcare staff visited each participant’s household and measured their blood pressure and blood sugar using the aforementioned portable measuring instrument. Hypertension was defined as systolic blood pressure ≥ 140 mm Hg or diastolic blood pressure ≥ 90 mm Hg [27]. Blood pressure measurements were recorded twice and the average was used in the final analysis. Diabetes was defined as a casual blood sugar level over 11.1 mmol/L. Height and weight were measured using standard procedure by the staff after completing the questionnaire survey.

The baseline questionnaire survey was performed by the research assistant after all participants provided verbally informed consent. The baseline survey included questions on the basic characteristics, namely, social demographics, literacy, daily medicine intake, comorbidity, experience of health checkup, and risk behaviors related to NCDs; the contents of the questionnaire for risk behavior were retrieved from the “STEPwise approach to NCD risk factor surveillance (STEPS)” developed and widely used by the World Health Organization (WHO). The survey items included risk behavior and beliefs related to NCDs and improvement in these items. The follow-up questionnaire survey included the same items.

### Outcomes

The predetermined primary outcome was the reduction in the prevalence proportions of hypertension and diabetes in the study sample. The secondary outcomes were changes in systolic blood pressure, casual blood sugar level, mean blood pressure ([systolic blood pressure—diastolic blood pressure] / 3 + diastolic blood pressure), the proportion of the participants who showed improved the behavior related to NCDs for each STEPS item, and proportion of overweight (25≦ Body Mass Index (BMI) < 30) and obesity (30≦ BMI).

### Statistical analysis

Regarding the primary outcome, confidential fixed-effects logistic model was used to identify the changes in the prevalence of hypertension and diabetes between pre- and post-intervention, for interventions one and two vs. control. For the secondary outcomes, multiple linear regression analysis was performed to identify the effect of intervention one and intervention two vs. control on systolic blood pressure, mean blood pressure, and blood glucose level between pre- and post-intervention. The linear regression model included the intervention group, sex, daily medicine use, and income as categorical independent variables and included age, education year, and testing result at baseline level as continuous independent variables. In addition, the proportions of the participants who showed improvements in the STEPS items were calculated. All analyses were performed using STATA IC15 (Lightstone, San Antonio, TX, USA, version 15).

## Results

### Participants

Overall, 600 participants completed the baseline survey and the follow-up survey. The baseline characteristics of sex, number of household members, medicine intake, comorbidity, BMI, and presence of hypertension and diabetes were similar among the intervention and control groups, but not those of age, education years, income and occupation (Table 1). The presence of hypertension at the baseline survey was much higher than the presence of diabetes. The proportions of the participants who used daily medicine were 26.5% in the first group, 25% in the second intervention group, and 24% in control group at the baseline survey (*p* = 0.84, Chi-squared test) and 29.5%, 25.5%, and 24.5%, respectively, at the follow-up survey (*p* = 0.48, Chi-squared test). No serious adverse events were associated with the interventions.

**Table 1:** Participant’s baseline characteristics in each intervention group (*N* = 600)

	Intervention 1 (*n* = 200)	Intervention 2 (*n* = 200)	Control (*n* = 200)	*p* value
Age (median [IQR])	48.5 [41–56]	45 [38–56]	45 [34.5–57.5]	0.002
Sex (female) (n (%))	93 (46.5)	97 (48.5)	90 (45.0)	0.78
Education years (median [IQR])	5 [0–9]	5 [0–9]	8 [0–10]	< 0.001
Income (n (%))				< 0.001
Below 200 USD	137 (68.5)	131 (65.5)	128 (64.0)	
200–400 USD	60 (30.0)	68 (34.0)	54 (27.0)	
400–600 USD	3 (1.5)	1 (0.5)	15 (7.5)	
600–800 USD	0 (0.0)	0 (0.0)	3 (1.5)	
Occupation (n (%))				< 0.001
Students	0 (0.0)	0 (0.0)	7 (3.5)	
Engaged in any work	1 (0.5)	1 (0.5)	3 (1.5)	
Agricultural and fishery	70 (35.0)	79 (39.5)	51 (25.5)	
Own business	39 (19.5)	47 (23.5)	42 (21.0)	
Housewife	80 (40.0)	54 (27.0)	77 (38.5)	
Employed	10 (5.0)	19 (9.5)	20 (10.0)	
House hold members (median [IQR])	5 [4–6]	4 [3–6]	4 [4–6]	0.142
Medicine taken (n (%))	53 (26.5)	50 (25.0)	48 (24.0)	0.85
Diagnosed hypertension by doctors (n (%))	37 (18.5)	38 (19.0)	41 (20.5)	0.87
Diagnosed diabetes by doctors (n (%))	21 (10.5)	23 (11.5)	17 (8.5)	0.60
Diagnosed dyslipidemia by doctors (n (%))	3 (1.5)	0 (0.0)	5 (2.5)	0.09
BMI (n (%))				0.82
Normal (18.5≦ BMI < 25)	117 (58.5)	106 (53.0)	116 (58.0)	
Underweight (BMI < 18.5)	18 (9.0)	19 (9.5)	15 (7.5)	
Overweight (25≦ BMI < 30)	52 (26.0)	57 (28.5)	57 (28.5)	
Obesity (30≦BMI)	13 (6.5)	18 (9.0)	12 (6.0)	
Hypertension (Founded this time or diagnosed previously) (n (%))	76 (38.0)	74 (37.0)	81 (40.5)	0.76
Diabetes (Founded this time or diagnosed previously) (n (%))	33 (16.5)	28 (14.0)	26 (13.0)	0.59

Chi-squared test was used for categorial variables and ANOVA was used for continuous variables when analyzing the difference between groups

*BMI* body mass index; *IQR* interquartile range

### Blood pressure outcomes

The mean systolic blood pressure (± standard error) was 130.2 ± 1.4 mm Hg in the first intervention group, 128.8 ± 1.3 mm Hg in the second intervention group, and 132.0 ± 1.4 mm Hg in the control group at the baseline survey; and 122.9 ± 0.9, 126.9 ± 1.0, and 127.2 ± 1.0 mm Hg, respectively, at the follow-up survey. The mean systolic blood pressure fell by 7.3 mm Hg (95% confidence interval [CI] 4.6–9.9) in the first intervention group, 1.9 mm Hg (95% CI − 0.5–4.2) in the second intervention group, and 4.7 mm Hg (95% CI 2.4–7.0) in the control group.

The median systolic blood pressure (IQR; interquartile range) was 128 (117–141) mm Hg in the first intervention group, 126 (117–140) mm Hg in the second intervention group, and 128 (119–141.5) mm Hg in the control group at the baseline survey; and 120 (110–130), 130 (120–136.5), and 130 (120–139) mm Hg, respectively, at the follow-up survey.

The prevalence of hypertension in the study sample decreased by 15% in the first intervention group, 6.5% in the second group, and 7.5% in the control group. The between-group differences in the prevalence of hypertension were not significant for interventions one and two versus control in the confidential fixed-effects logistic model (Table 2). The between-group differences in the decline in systolic blood pressure after the intervention were significant on multiple linear regression analysis for intervention one versus control (*p* = 0.001), but not for intervention two versus control (*p* = 0.21) (Table 3). The between-group differences in the decline in the mean blood pressure were significant for intervention one versus control (*p* < 0.001), but not for intervention two versus control (*p* = 0.75) (Additional file 1: Table S1). Distribution of systolic blood pressure before intervention by group of past medical history was shown in Additional file 1: Figure S3. Systolic blood pressure each group of pre diagnosed and classification was shown Additional file 1: Table S2. In addition, we performed multiple linear regression analysis for the decrease in systolic blood pressure between pre- and post-intervention one considering interaction. The result was not changed so much after considering interaction between education and sex, sex and income. (Additional file 1: Tables S3 and S4).

**Table 2:** Results of effectiveness of each intervention using confidential fixed-effects logistic model

	Pre intervention n (%)	Post intervention n (%)	Difference of pre/post (%)	Odds ratio [95% confidential interval]^+^	*p* value^+^
Outcome: the presence of hypertension					
Intervention 1	62 (31.0)	32 (16.0)	− 15	0.58 [0.25–1.34]	0.20
Intervention 2	58 (29.0)	45 (22.5)	− 6.5	1.09 [0.49–2.44]	0.84
Control	65 (32.5)	50 (25.0)	− 7.5		
Outcome: the presence of diabetes					
Intervention 1	23 (11.5)	11 (5.5)	− 6	1.90 [0.28–12.87]	0.51
Intervention 2	13 (6.5)	3 (1.5)	− 5	1.58 [0.20–12.79]	0.67
Control	23 (11.5)	6 (3.0)	− 8.5		

Hypertension was defined as systolic blood pressure ≥ 140 mm Hg or diastolic blood pressure ≥ 90 mm Hg at pre-test. Diabetes was defined as casual blood sugar level over 11.1 mmol/L at pre-test

^+^ Outcome was increased proportion of hypertension and diabetes by intervention verses control

**Table 3:** Multiple linear regression analysis of the decrease of systolic blood pressure between pre- and post-intervention

	B	Standard error of B	Beta	*p* value
Intervention 1 (base: control)	4.175	1.267	0.118	0.001
Age	− 0.157	0.062	− 0.106	0.012
Sex (base: male)	1.161	1.272	0.033	0.36
Education [year]	− 0.311	0.160	− 0.082	0.05
Daily medicine (base: none)	− 8.658	1.581	− 0.213	< 0.001
Income (base: Below 200 USD/month)				
200–400 USD / month	− 0.315	1.474	− 0.008	0.83
400–600 USD / month	− 4.388	3.093	− 0.052	0.157
600–800 USD / month	1.401	7.129	0.007	0.84
Mean blood pressure at pre test	1.090	0.052	0.776	< 0.001
Intervention 2 (base: control)	− 1.471	1.173	− 0.044	0.21
Age	− 0.184	0.053	− 0.141	0.001
Sex (base: male)	− 0.582	1.203	− 0.018	0.63
Education [year]	− 0.175	0.151	− 0.049	0.25
Daily medicine (base: none)	− 11.544	1.509	− 0.300	< 0.001
Income (base: under 200 USD/month)				
200–400 USD / month	− 2.210	1.383	− 0.062	0.111
400–600 USD / month	− 7.665	3.063	− 0.091	0.013
600–800 USD / month	− 0.248	6.661	− 0.001	0.97
Mean blood pressure at pre test	1.055	0.050	0.785	< 0.001

*B* coefficient, Beta = adjusted coefficient

### Blood glucose outcomes

The mean blood glucose (± standard error) was 8.2 ± 0.3 mmol/L in the first intervention group, 6.6 ± 0.2 mmol/L in the second intervention group, and 7.6 ± 0.3 mmol/L in the control group at the baseline and 7.2 ± 0.2, 6.7 ± 0.1, and 6.7 ± 0.1 mmol/L, respectively, at the follow-up survey. The mean blood glucose level had decreased—by 1.1 mmol/L (95% CI 0.6–1.6) in the first intervention group, increased by 0.1 mmol/L (95% CI − 0.3–0.5) in the second intervention group, and decreased by 0.9 mmol/L (95% CI 0.4–1.4) in the control group.

The proportion of the presence of diabetes in the sample decreased by 6% in the first intervention group, 5% in the second intervention group, and 8.5% in the control group. The between-group differences in the prevalence of diabetes were not significant for both intervention one versus control and intervention two verses control on the confidential fixed-effects logistic model (Table 2). The between-group differences in the decrease in the blood glucose level after the intervention were not significant on multiple linear regression analysis (Table 4). Distribution of blood sugar level before intervention by group of past medical history was shown in Additional file 1: Figure S3. Blood sugar-level each group of pre-diagnosed and classification is shown in Additional file 1: Table S5.

**Table 4:** Multiple linear regression analysis for the decrease of blood sugar between pre- and post-intervention

	B	Standard error of B	Beta	*p* value
Intervention 1 (base: control)	− 0.341	0.193	− 0.049	0.078
Age	− 0.012	0.009	− 0.040	0.22
Sex (base: male)	0.335	0.195	0.048	0.087
Education [year]	− 0.051	0.025	− 0.069	0.037
Daily medicine (base: none)	− 0.729	0.248	− 0.091	0.003
Income (base: under 200 USD/month)				
200–400 USD / month	0.194	0.225	0.025	0.39
400–600 USD / month	0.466	0.472	0.028	0.32
600–800 USD / month	− 2.695	1.089	− 0.066	0.014
Blood sugar at pre test	0.738	0.024	0.880	< 0.001
Intervention 2 (base: control)	− 0.254	0.141	− 0.040	0.072
Age	− 0.007	0.006	− 0.029	0.25
Sex (base: male)	0.337	0.143	0.052	0.019
Education [year]	− 0.044	0.018	− 0.064	0.014
Daily medicine (base: none)	− 1.312	0.180	− 0.176	< 0.001
Income (base: under 200 USD/month)				
200–400 USD / month	0.084	0.165	0.012	0.61
400–600 USD / month	0.149	0.364	0.009	0.68
600–800 USD / month	− 2.573	0.793	− 0.069	0.001
Blood sugar at pre test	0.871	0.021	0.940	< 0.001

*B* coefficient, Beta = adjusted coefficient

### Other secondary outcomes

**Fig. 2:**
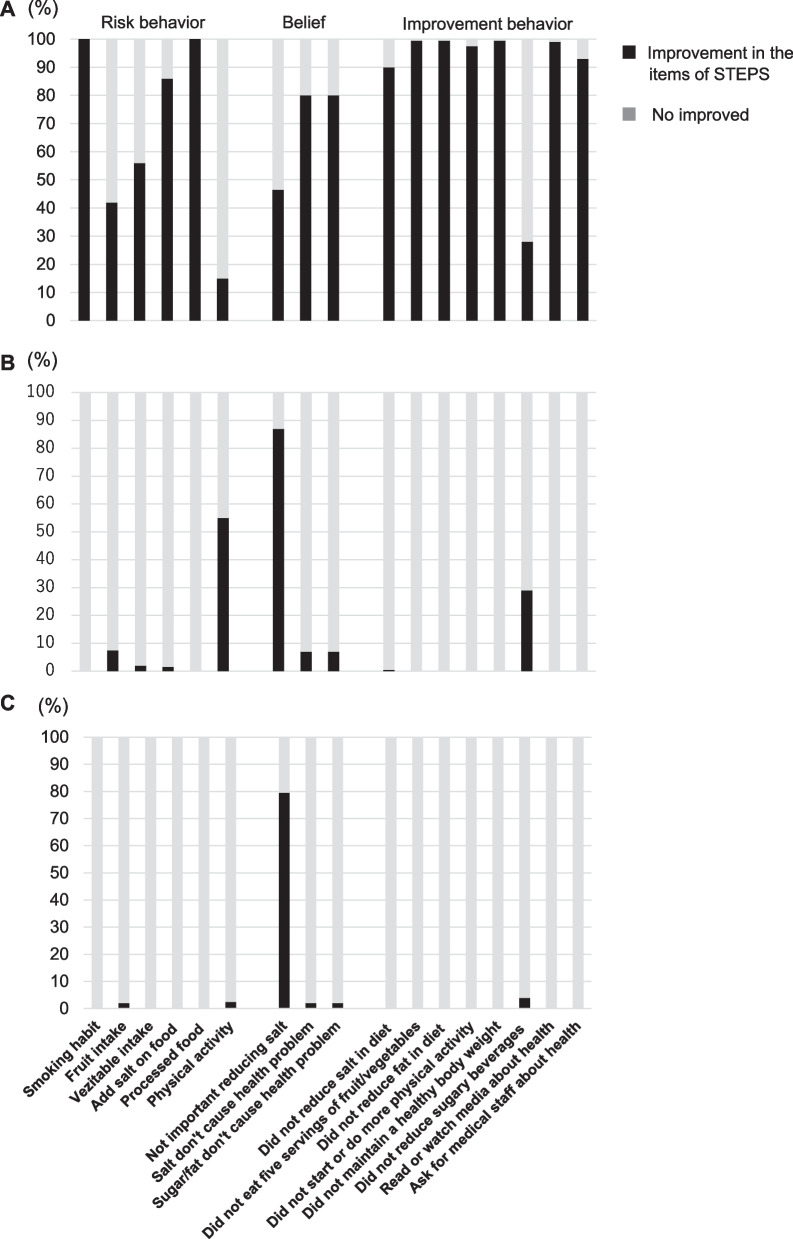
Proportions of the participants who showed improved behaviors for each STEPwise approach to NCD risk factor surveillance (STEPS) item**.** The survey consisted of 17 items under 3 categories: risk behavior (6 items), belief (3 items), and improvement in behavior (8 items). If a participant’s behavior and belief improved, the participant was classified under “Improvement in the items of STEPS.” If the behavior and belief did not improve, the participant was classified under “No improvement” **A** intervention 1 group, **B** intervention 2 group, **C** control group

The proportion of the participants who showed improved behavior toward NCDs for each STEPS item is shown in Fig. 2. The participants in the first intervention group showed overall improved behavior, but those of the second intervention group did not. The prevalence of obesity (BMI > 25) decreased by 2.5% in the first intervention group and by 2.5% in the second intervention group and increased by 0.5% in the control group.

## Discussion

Seeking strategy toward NCDs with community-based educational approached was essential in resource-limited setting, especially rural areas of LMICs. The present interventional study examined the effectiveness of a community-based, simple educational program run by non-healthcare trained staff for several outcomes associated with NCDs.

The educational intervention was found to be effective in reducing systolic blood pressure; however, it was not effective to decrease the proportion of hypertension. Systolic blood pressure and mean blood pressure had reduced significantly after the intervention in the first (strong) intervention group. However, no significant change was observed in the prevalence of hypertension in the sample. Similar to our results, previous studies have shown that community-based interventions improved blood pressure outcomes [7, 18, 20]. For reducing the prevalence of hypertension, community-based educational interventions should be implemented actively; strategies involving non-healthcare staff and community-based participation should be adopted in resource-limited settings.

The weak intervention group did not show improvements in hypertension, diabetes, and risk behavior. This result is in contrast with that of a previous intervention study, without a control group, where the use of a checklist poster for pesticide protective behavior significantly changed the behavior among farmers in the region [26]. This finding indicates the need for more frequent and elaborate interventions than the weak intervention to improve blood pressure and risk behavior related to NCDs.

In this study, the interventions were not effective in improving the primary and secondary outcomes associated with diabetes. However, in a previous study, an intervention provided by the community health worker or pharmacist improved the outcomes associated with diabetes [8, 28]. Disease-oriented and well-planned intervention strategies are required for improving the outcome associated with diabetes.

Multiple liner regression analysis showed that those who were young people, those without medications, those with few education years, and those with high blood pressure at the baseline significantly reduced the blood pressure outcome among intervention one group versus control group. The proportion of participants with daily medication did not change substantially between pre- and post-intervention. The finding that blood pressure decreased among those with high blood pressure at the baseline and without daily medication supports the usefulness of community-based interventions for hypertension-related outcomes. Moreover, considering that the interventions was effective among younger residents and those with low levels of education, such strategies should be actively implemented among these populations.

The present study has some limitations that prevent the generalization of the results. First, it was not a clustered randomized control trial because the number of clusters was small. Well-designed clustered randomized control trials are required to generate strong evidence. Second, some of the baseline characteristics of the participants were different among groups. Third, the outcome of diabetes was based on casual blood sugar, measured using a portable measuring instrument; fasting blood sugar or HbA1c would have provided more reliable results. Fourth, only 59.8% of the participants could read and write, and this affected the intervention procedure. The local non-healthcare staff struggled to explain the interventions to these participants. Fifth, sample size was not calculated based on statistical formula before survey, and sample size was small. Sixth, study participants were only those who were interested in the study and were randomly selected; thus the study population was subjected to the restriction. Seventh, seminar videos and face to face seminar reached only 60% and 40% participants respectively. Eighth, we choose proportion of hypertension and diabetes as primary outcome, rather than more common outcome, systolic blood pressure and blood glucose. Finally, this study was conducted on a selected group of population in selected rural areas of a LMIC; more studies are required in different settings in different LMICs for the validation of the findings. Despite these limitations, the present study was valuable on large-scale interventional study with a control group for the educational intervention toward NCDs by non-healthcare staff in the rural community in Asian LMICs.

## Conclusions

Community-based educational interventions provided by non-healthcare staff reduced the average of systolic pressure; however, it was not effective to decrease the proportion of the presence of hypertension and diabetes. Well-designed community-based educational interventions should be implemented frequently to reduce the prevalence of NCDs in rural areas of LMICs.

### Supplementary Information

**Additional file 1: Figure S1.** Lifestyle checklist poster on the wall to prevent noncommunicable diseases. Participants were required to stick the poster on their house wall. The poster was translated in the local language. Some modifications were performed before translation for considering local culture. **Figure S2.** Semi-advice paper. Grouping was conducted using baseline data by latent class analysis. The advice paper was translated in the local language. **Figure S3.** Systolic blood pressure and blood sugar level before intervention by group of past medical history. Systolic blood pressure (B) blood sugar. **Table S1.** Multiple linear regression analysis for the decrease in mean blood pressure between pre- and post-intervention. **Table S2.** Systolic blood pressure each group of pre-diagnosed and classification. **Table S3.** Education years and Income by sex. **Table S4.** Multiple linear regression analysis for the decrease in systolic blood pressure between pre- and post-intervention one considering interaction. **Table S5.** Blood sugar each group of pre-diagnosed and classification.

## Data Availability

The data relating to this manuscript are available upon reasonable request.

## Abbreviations

NCDs: Non-communicable diseases
LMICs: Low- and middle-income countries
