# Supplementary material for: Community-based intervention for managing hypertension and diabetes in rural Bangladesh
Source: Trop Med Health. 2024 Jan 24;52:13. doi: 10.1186/s41182-023-00574-0 (PMC10807096; doi:10.1186/s41182-023-00574-0)
Supplement: Supplementary file 1 — Additional file 1: Figure S1. Lifestyle checklist poster on the wall to prevent noncommunicable diseases. Participants were required to stick the poster on their house wall. The poster was translated in the local language. Some modifications were performed before translation for considering local culture. Figure S2. Semi-advice paper. Grouping was conducted using baseline data by latent class analysis. The advice paper was translated in the local language. Figure S3. Systolic blood pressure and blood sugar level before intervention by group of past medical history. Systolic blood pressure (B) blood sugar. Table S1. Multiple linear regression analysis for the decrease in mean blood pressure between pre- and post-intervention. Table S2. Systolic blood pressure each group of pre-diagnosed and classification. Table S3. Education years and Income by sex. Table S4. Multiple linear regression analysis for the decrease in systolic blood pressure between pre- and post-intervention one considering interaction. Table S5. Blood sugar each group of pre-diagnosed and classification. [file 41182_2023_574_MOESM1_ESM.docx]

**Figure S1. Lifestyle checklist poster on the wall to prevent noncommunicable diseases**

Participants were required to stick the poster on their house wall. The poster was translated in the local language. Some modifications were performed before translation for considering local culture.


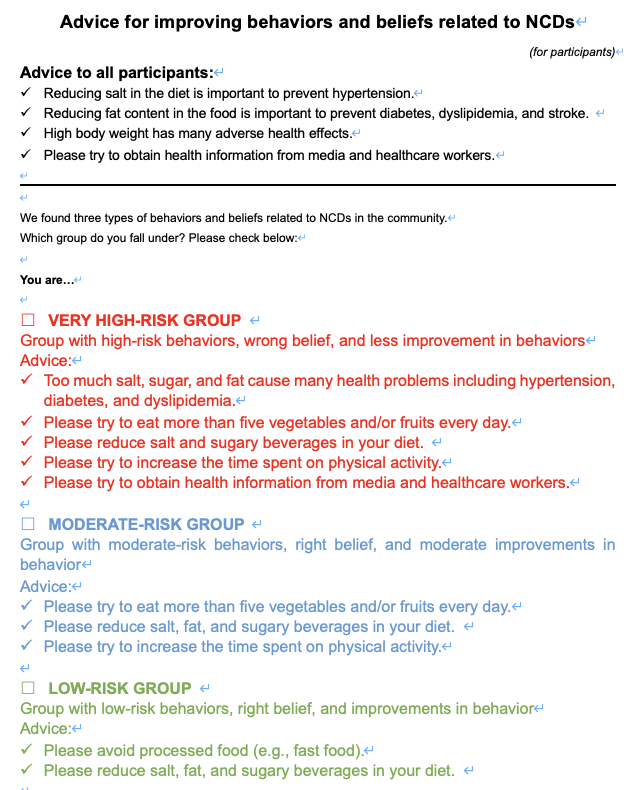


**Figure S2. Semi-advice paper**

Grouping was conducted using baseline data by latent class analysis. The advice paper was translated in the local language.

**Table S1. Multiple linear regression analysis for the decrease in mean blood pressure between pre- and post-intervention**

|  | B | Standard error of B | Beta | *p*-value |
| --- | --- | --- | --- | --- |
| Intervention 1 (base: control) | 3.681 | 0.777 | 0.151 | <0.001 |
| Age | -0.217 | 0.038 | -0.212 | <0.001 |
| Sex (base: male) | 0.939 | 0.780 | 0.038 | 0.23 |
| Education [year] | -0.195 | 0.098 | -0.075 | 0.048 |
| Daily medicine (base: none) | -7.231 | 0.970 | -0.257 | <0.001 |
| Income (base: Below 200 USD/month) |  |  |  |  |
| 200–400 USD / month | -1.134 | 0.904 | -0.042 | 0.21 |
| 400–600 USD / month | -1.837 | 1.897 | -0.031 | 0.33 |
| 600–800 USD / month | 4.828 | 4.372 | 0.034 | 0.27 |
| Mean blood pressure at pre-test | 0.821 | 0.032 | 0.845 | <0.001 |
| Intervention 2 (base: control) | 0.232 | 0.723 | 0.010 | 0.75 |
| Age | -0.216 | 0.033 | -0.238 | <0.001 |
| Sex (base: male) | 0.446 | 0.742 | 0.019 | 0.55 |
| Education [year] | -0.232 | 0.093 | -0.094 | 0.013 |
| Daily medicine (base: none) | -10.059 | 0.931 | -0.375 | <0.001 |
| Income (base: under 200 USD/month) |  |  |  |  |
| 200–400 USD / month | -1.643 | 0.853 | -0.066 | 0.055 |
| 400–600 USD / month | -1.598 | 1.890 | -0.027 | 0.40 |
| 600–800 USD / month | 5.142 | 4.109 | 0.038 | 0.21 |
| Mean blood pressure at pre-test | 0.795 | 0.031 | 0.848 | <0.001 |

B = coefficient, Beta = adjusted coefficient

**Figure S3. Systolic blood pressure and blood sugar level before intervention by group of past medical history**

Systolic blood pressure (B) blood sugar

**Table S2. Systolic blood pressure each group of pre-diagnosed and classification**

|  | Diagnosed and well controlled | |  | Diagnosed and bad controlled | |  | Not diagnosed and  sBP < 130 | |  | Not diagnosed and  130≦ sBP <140 | |  | Not diagnosed and  140 ≦ sBP | |
| --- | --- | --- | --- | --- | --- | --- | --- | --- | --- | --- | --- | --- | --- | --- |
|  | Mean | SD |  | Mean | SD |  | Mean | SD |  | Mean | SD |  | Mean | SD |
| Pre-Intervention |  |  |  |  |  |  |  |  |  |  |  |  |  |  |
| Intervention 1 | 125.4 | 11.5 |  | 154.3 | 9.8 |  | 115.3 | 8.0 |  | 133.7 | 2.7 |  | 156.3 | 16.0 |
| Intervention 2 | 124.9 | 8.8 |  | 160.0 | 16.3 |  | 116.5 | 9.0 |  | 133.8 | 2.8 |  | 147.1 | 7.1 |
| Control | 128.2 | 7.0 |  | 164.4 | 18.6 |  | 118.0 | 8.1 |  | 133.9 | 2.9 |  | 150.2 | 10.8 |
| Post-Intervention |  |  |  |  |  |  |  |  |  |  |  |  |  |  |
| Intervention 1 | 127.1 | 15.2 |  | 136.2 | 15.3 |  | 118.5 | 10.9 |  | 123.1 | 9.0 |  | 125.0 | 11.2 |
| Intervention 2 | 138.1 | 10.5 |  | 145.1 | 11.1 |  | 121.0 | 10.9 |  | 126.0 | 12.5 |  | 129.4 | 13.8 |
| Control | 142.9 | 6.2 |  | 146.4 | 7.8 |  | 119.6 | 9.8 |  | 123.9 | 9.8 |  | 131.0 | 9.3 |

sBP = systolic blood pressure (mm Hg), well control means sBP < 140, SD = standard deviation

**Table S3. Education years and Income by sex**

|  | Male | Female |
| --- | --- | --- |
| Education years |  |  |
| 0–5 years education | 156 (47.6) | 172 (52.4) |
| 6–10 years education | 86 (49.4) | 88 (50.6) |
| Over 10 years education | 78 (79.6) | 20 (20.4) |
| Income |  |  |
| Below 200 USD/month | 188 (47.5) | 208 (52.5) |
| 200–400 USD / month | 120 (65.9) | 62 (34.1) |
| 400–600 USD / month | 9 (47.4) | 10 (52.6) |
| 600–800 USD / month | 3 (100.0) | 0 (0.0) |

**Table S4. Multiple linear regression analysis for the decrease in systolic blood pressure between pre- and post-intervention one considering interaction**

|  | B | Standard error of B | Beta | *p*-value |
| --- | --- | --- | --- | --- |
| Intervention 1 (base: control) | 4.23 | 1.36 | 0.12 | 0.002 |
| Age | -0.28 | 0.06 | -0.19 | <0.001 |
| Sex (base: male) | 0.12 | 1.82 | 0.00 | 0.95 |
| Education (base: 0–5years education) |  |  |  |  |
| 6–10 years education | -1.89 | 2.19 | -0.05 | 0.39 |
| Over 10 years education | -5.76 | 2.63 | -0.13 | 0.029 |
| Income (base: Below 200 USD/month) |  |  |  |  |
| 200–400 USD / month | 1.24 | 2.12 | 0.03 | 0.56 |
| 400–600 USD / month | -3.87 | 4.79 | -0.05 | 0.42 |
| 600–800 USD / month | 3.62 | 7.65 | 0.02 | 0.64 |
| Mean blood pressure at pre test | 1.03 | 0.05 | 0.73 | <0.001 |
| Interaction (Sex × Education) |  |  |  |  |
| Female × 6–10 years education | -0.32 | 3.01 | -0.01 | 0.92 |
| Female **×** Over 10 years education | 5.62 | 4.57 | 0.06 | 0.22 |
| Interaction (Sex × Income) |  |  |  |  |
| Female × 200–400 USD / month | -2.58 | 3.14 | -0.04 | 0.41 |
| Female × 400–600 USD / month | -2.88 | 6.68 | -0.03 | 0.67 |
| Female × 600–800 USD / month | NA | NA | NA | NA |

B = coefficient, Beta = adjusted coefficient

**Table S5. Blood sugar each group of pre-diagnosed and classification**

|  | Diagnosed and well control | |  | Diagnosed and bad control | |  | Not diagnosed and  blood sugar < 10 | |  | Not diagnosed and  10 ≦ blood sugar < 11.1 | |  | Not diagnosed and  11.1 ≦ blood sugar | |
| --- | --- | --- | --- | --- | --- | --- | --- | --- | --- | --- | --- | --- | --- | --- |
|  | Mean | SD |  | Mean | SD |  | Mean | SD |  | Mean | SD |  | Mean | SD |
| Pre-Intervention |  |  |  |  |  |  |  |  |  |  |  |  |  |  |
| Intervention 1 | 9.2 | 2.2 |  | 19.4 | 5.8 |  | 6.5 | 1.4 |  | 10.4 | 0.3 |  | 17.3 | 4.6 |
| Intervention 2 | 6.9 | 1.5 |  | 16.2 | 4.7 |  | 5.9 | 1.2 |  | 10.2 | 0.2 |  | 14.7 | 4.5 |
| Control | 7.7 | 2.2 |  | 17.6 | 4.9 |  | 6.3 | 1.3 |  | 10.3 | 0.2 |  | 14.9 | 4.5 |
| Post-Intervention |  |  |  |  |  |  |  |  |  |  |  |  |  |  |
| Intervention 1 | 9.3 | 1.9 |  | 10.8 | 2.8 |  | 6.5 | 1.0 |  | 6.8 | 0.7 |  | 10.3 | 8.3 |
| Intervention 2 | 9.2 | 1.6 |  | 9.3 | 2.1 |  | 6.4 | 1.1 |  | 6.2 | 0.3 |  | 6.7 | 0.9 |
| Control | 12.6 | 2.2 |  | 10.5 | 2.8 |  | 6.3 | 0.9 |  | 7.1 | 0.7 |  | 6.3 | 0.3 |

Blood sugar (mmol/L), well control means blood sugar < 11.1, SD = standard deviation
